# Experimentally obtained and computer-simulated X-ray non-coplanar 18-beam pinhole topographs for a silicon crystal

**DOI:** 10.1107/S2053273319002936

**Published:** 2019-04-30

**Authors:** Kouhei Okitsu, Yasuhiko Imai, Yoshitaka Yoda

**Affiliations:** a Nano-Engineering Research Center, Institute of Engineering Innovation, Graduate School of Engineering, The University of Tokyo, 2-11-16 Yayoi, Bunkyo-ku, Tokyo 113-8656, Japan; b Japan Synchrotron Radiation Research Institute, SPring-8, 1-1-1 Kouto, Mikazuki-cho, Sayo-gun, Hyogo 679-5198, Japan

**Keywords:** X-ray diffraction, dynamical theory, multiple reflection, *n*-beam reflection, phase problem, protein crystallography

## Abstract

Non-coplanar 18-beam X-ray pinhole topographs for a silicon crystal were computer simulated by fast Fourier transforming the X-ray rocking amplitudes that were obtained by solving the *n*-beam (*n* = 18) Ewald–Laue dynamical theory (E-L&FFT method). They were in good agreement with the experimentally obtained images captured using synchrotron X-rays. From this result and further consideration based on it, it has been clarified that the X-ray diffraction intensities when *n* X-ray waves are simultaneously strong in the crystal can be computed for any *n* by using the E-L&FFT method.

## Abstract 2

Experimentally obtained non-coplanar 18-beam pinhole topographs were compared with computer simulations based on the Ewald–Laue theory.

## 1. Introduction   

**Figure 1:**
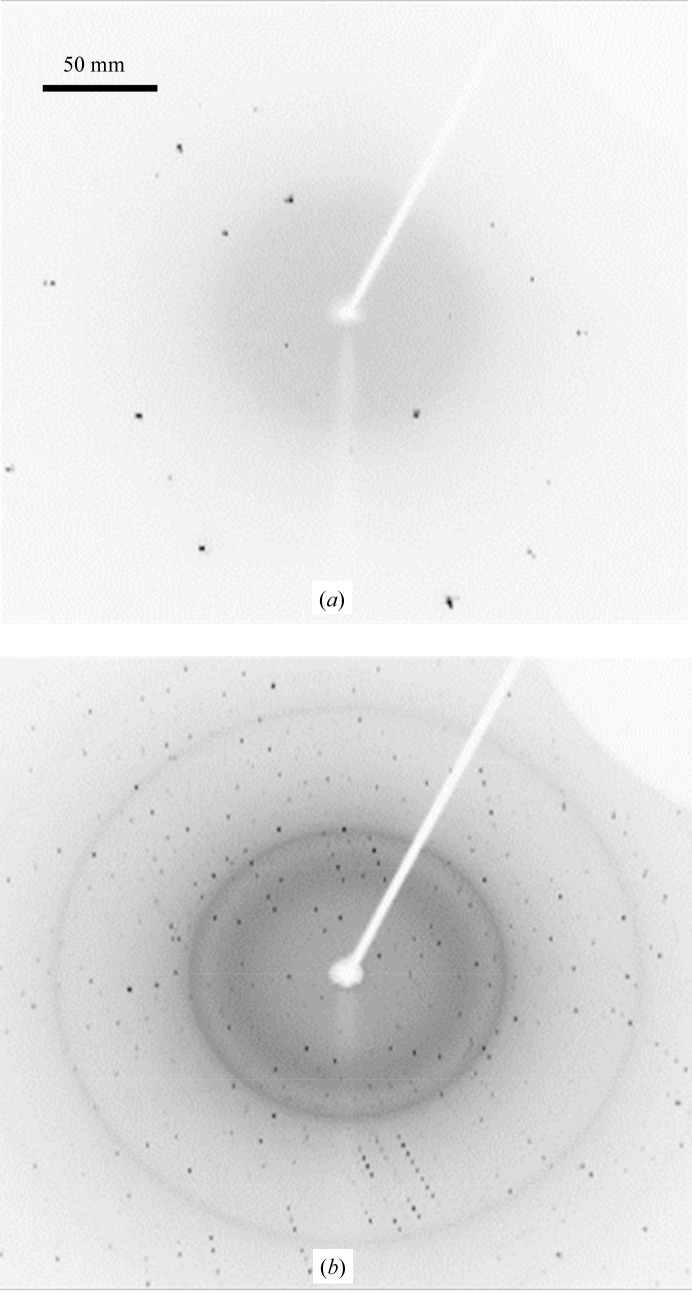
Diffraction spots for (*a*) a sucrose (small molecular weight) crystal and (*b*) a hen egg-white lysozyme (protein) crystal taken on the imaging plate (IP) of a Rigaku Micro7 HFM-AXIS7 diffractometer. The distance between the crystal and the IP was 150 mm. The IP was exposed for 60 s by oscillating the crystal in the range of 0.1° for both (*a*) and (*b*).

The present authors have reported coplanar eight-beam pinhole topographs experimentally obtained and computer simulated by fast Fourier transforming (FFT) the rocking amplitudes calculated based on the *n*-beam Ewald–Laue (E-L) theory. This technique (E-L&FFT simulation) was reported by Kohn & Khikhlukha (2016 ▸) and Kohn (2017 ▸). In Okitsu *et al.* (2019 ▸), it was shown that the E-L&FFT simulation can also be performed for a case where the X-rays do not exit from a single plane (hereafter this paper is denoted as O *et al.* 2019). Furthermore, the feasibility of calculating the X-ray intensities diffracted from a crystal that has plural facets, as shown in Fig. 9 of O *et al.* (2019), was discussed. In addition to this, if the E-L&FFT simulation could be performed even for a case where 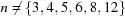 (non-coplanar case), the intensities of X-ray diffraction spots from a lysozyme (protein) crystal as shown in Fig. 1 ▸(*b*) could be calculated. Here a large number (over 200) of reflected X-ray beams are simultaneously strong.

## 2. Experimental   

**Figure 2:**
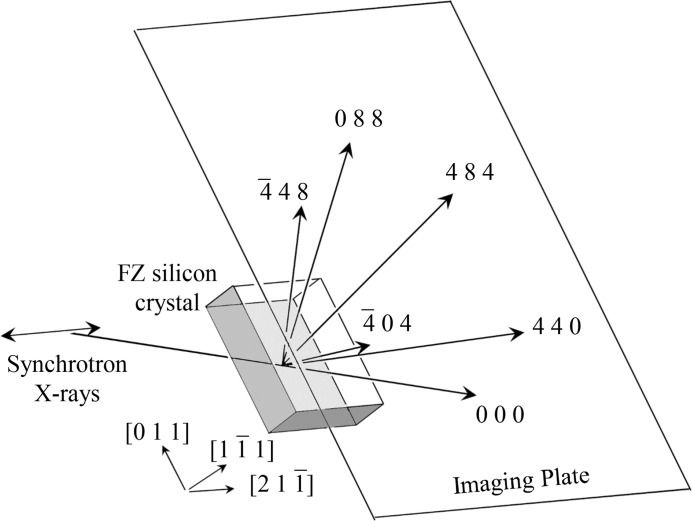
Schematic drawing of the experimental setup. The horizontally polarized synchrotron X-rays were incident on a [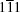]-oriented floating-zone (FZ) silicon crystal with a thickness of 10.0 mm such that the six beams are simultaneously strong. The angle of the monochromator was adjusted such that the photon energy of the X-rays was 22.0 keV. However, the practical value of the photon energy was considered to be marginally different from this value. An IP was placed 24 mm behind the crystal.

Fig. 2 ▸ shows the experimental arrangement. The horizontally polarized synchrotron X-rays at BL09XU of SPring-8 were monochromated to be 22.0 keV. The phase retarder system was not used in the present experiment. The beam size was limited to 25 × 25 µm. The goniometer system on which a [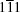]-oriented floating-zone (FZ) silicon crystal was mounted was adjusted such that the 000 forward-diffracted (FD) and 440, 484, 088, 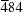 and 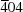 transmitted-reflected (TR) X-rays are simultaneously strong; this was achieved by monitoring the 000 FD, 440 and 484 TR X-rays with PIN photodiodes. The thickness of the crystal was 10.0 mm. An imaging plate (IP) was placed 24 mm behind the crystal such that the surface of the IP was parallel to the exit surface of the crystal.

**Figure 3:**
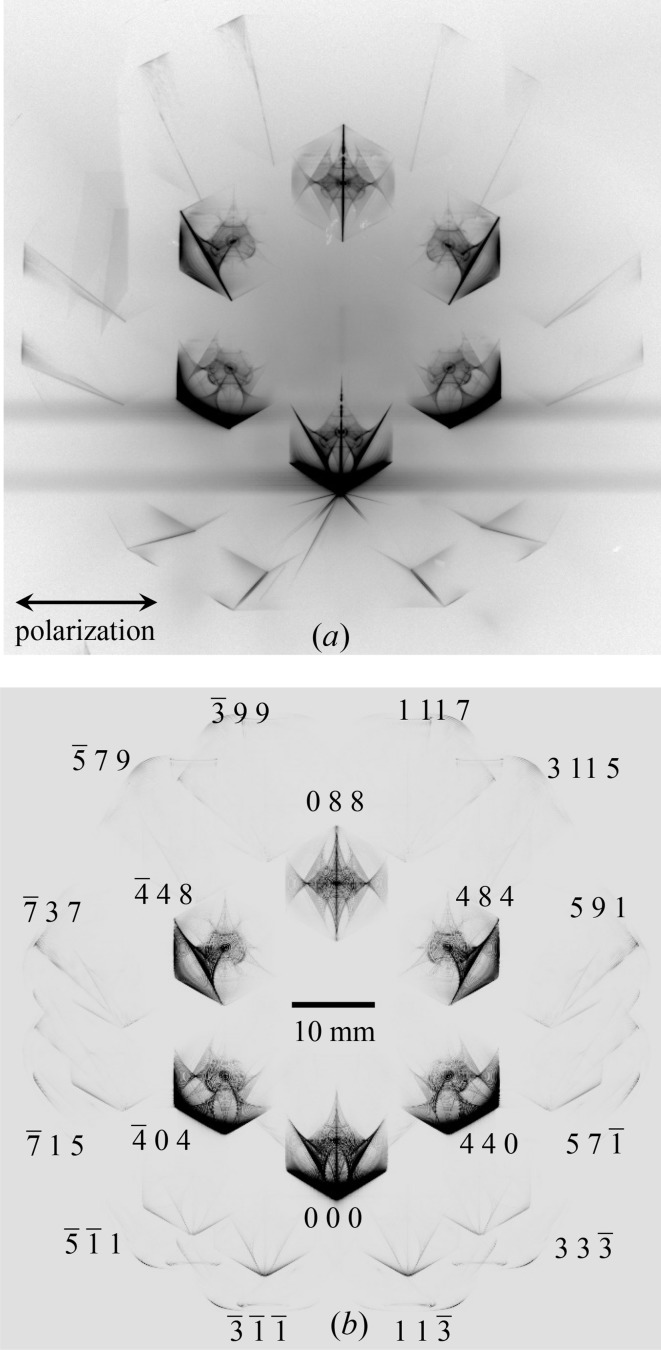
(*a*) Experimentally obtained and (*b*) E-L&FFT simulated 18-beam pinhole topographs. (*b*) was obtained by the E-L&FFT simulation under an assumption of an incidence of X-rays with a photon energy *E* = 21.98415 keV (Δ*E* = *E* − *E*_0_ = −0.25 eV, where *E*_0_ = 21.98440 keV).

In addition to the hexagonal six-beam topograph images, a further 12 images surrounding them were found on the IP as shown in Fig. 3 ▸(*a*). The exposure time was 300 s.

## 3. Computer simulation   

**Figure 4:**
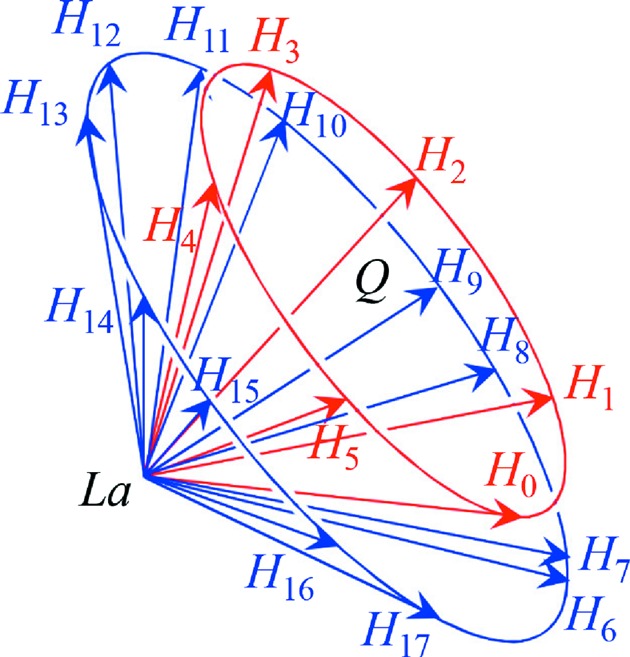
Six reciprocal-lattice nodes are on a red circle in reciprocal space. Outside of this circle, a blue circle was observed on which 12 reciprocal-lattice nodes were present. *Q* is the centre of the red circle.

The length of the wavevector *K* (= 1/λ, where λ is the wavelength in vacuum) was calculated to be 1.7702394 Å^−1^ for a photon energy of 22.0 keV. The position of the Laue point *La* whose distance from reciprocal-lattice nodes 000, 440, 484, 088, 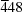 and 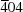 was an identical value *K*, was calculated on a computer. From Fig. 3 ▸(*a*), other reciprocal-lattice nodes were likely to exist in the vicinity of the surface of the Ewald sphere; that is, their distance from *La* was approximately 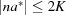, *i.e.* 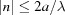 is the sufficient condition for a reciprocal-lattice node with indices *hkl* to exist on the surface of the Ewald sphere. Here, *a* is the lattice constant of the silicon crystal, 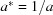 and 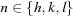. Because 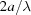 was calculated to be 18.21, the distances of reciprocal-lattice nodes with indices *hkl* from *La* were calculated in the range of 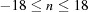. Then, in addition to the six reciprocal-lattice nodes, others with 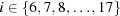 were observed, as summarized in Table 1 ▸. Here, *i* is the ordinal number of the reciprocal-lattice node in the first column of Table 1 ▸. Then, all topograph patterns surrounding 000 FD, 440, 484, 088, 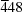 and 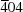 TR images have been indexed as shown in Fig. 3 ▸(*b*). For obtaining this figure, a photon energy of 21.98415 keV was assumed. It was observed that the *i*th reciprocal-lattice nodes (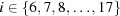) were on another circle (drawn as a blue circle in Fig. 4 ▸) outside the circle (drawn as a red circle whose centre is *Q* in Fig. 4 ▸) on which the inner six reciprocal-lattice nodes are present. For these 18 FD or TR X-ray beams with indices 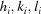 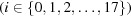, the Bragg reflection angle 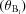, 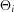, 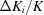, 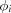 and 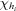 were calculated and are summarized in Table 1 ▸. 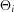 is the angle spanned by 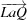 and 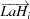 where 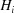 is the *i*th-numbered reciprocal-lattice node in Fig. 4 ▸. 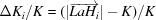. 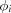 is the inclination angle of 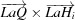 from 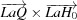.

**Figure 5:**
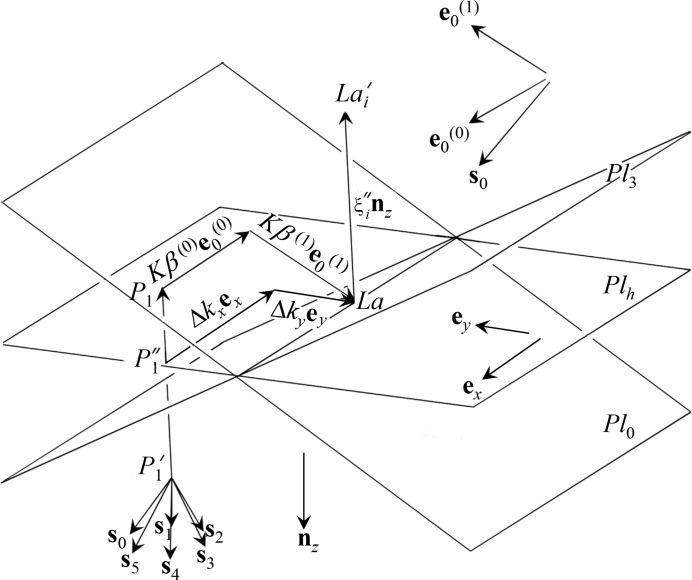
Geometry around the Laue point *La*. 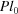 and 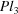 are planes whose distance from 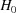 and 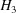 is *K*. 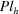 is a plane normal to 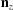 (downward surface normal). The Laue point *La* and point 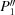 exist on 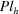. 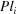 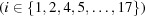 were not drawn for simplicity. 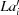 is a point whose distance from 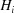 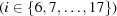 is *K*. 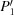 is the initial point of the wavevector of the Bloch wave. 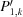 that appears in equation (14) in O *et al.* (2019) is the *k*th-numbered 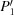, *i.e.* the initial point of the wavevector of the *k*th-numbered Bloch wave where 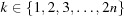.

Fig. 5 ▸ is a drawing around the Laue point *La*. Here, let another Laue point 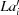 be defined in the vicinity of *La* as shown in Fig. 5 ▸ such that 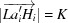. Because *Q* is the circumcentre of the normal hexagon whose vertices are 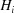 (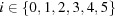) as shown in Fig. 4 ▸, 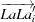 is evidently 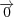 for 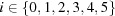 and is an identical vector in the direction of 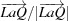 (= 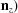 for 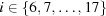. Here, let 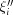 be defined such that 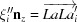 as shown in Fig. 5 ▸. 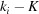 on the left-hand side of equation (4) in O *et al.* 2019 can be described as follows: 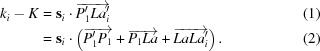
Because 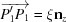, 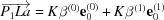 and 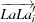 = 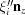, where 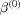 and 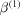 are the two-dimensional angular deviation of 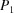 from *La* as shown in Fig. 5 ▸. Therefore, equation (2) can be modified as follows: 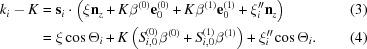
The polarization factors *C* and *S* are defined as 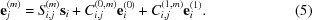
In the present 18-beam case, 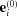 was defined to be 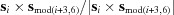 for 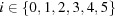 and to be 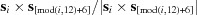 for 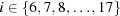. 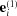 was defined to be 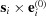 for 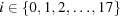.

Laue’s fundamental equation of the dynamical theory (von Laue, 1931 ▸; Authier, 2005 ▸) restricts the amplitude and wavevector of the Bloch wave as follows: 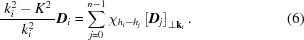
Here 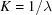, where λ is the wavelength of the X-rays in vacuum, and 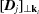 is the component vector of 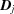 perpendicular to 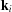. By applying the approximation 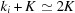, equation (6) becomes
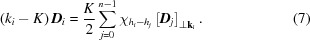
Substituting equation (4) into equation (7), the following equation can be obtained:
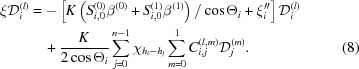
Equation (8) is represented by using a vector and a matrix as follows: 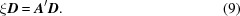
Here 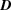 is a 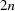-order column vector and 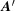 is a 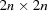 matrix whose element in the *p*th row 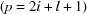 and *q*th column 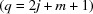 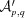 is given by 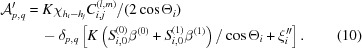
Here, 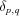 is the Kronecker delta. Moreover, for the present 18-beam case, the procedure described by equations (10)–(16) in O *et al.* 2019 can be used to solve the eigenvalue problem of equations (9) and (10). The values of 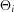, 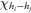 and 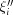 listed in Tables 1 ▸ and 2 ▸ were used.

Furthermore, for the FFT to compute the E-L&FFT topographs, the description using equations (17)–(20) in O *et al.* 2019 can also be applied to the present 18-beam case. The FFT in equation (20) in O *et al.* 2019 was carried out with *L* = 50 mm and *N* = 4096.

It required 1080 s (890 s for solving the eigenvalue problem, 20 s for FFT and 170 s for writing the topographs to the hard disk) to obtain the 18 topograph images shown in Fig. 3 ▸(*b*) using one node (Intel Xeon E5-2680v3) of the supercomputer system ‘Sekirei’ of the Institute of Solid State Physics of the University of Tokyo. The calculation to solve the eigenvalue problem for a 36 × 36 matrix was several times as time-consuming as the coplanar eight-beam case solving the eigenvalue problem described with two 16 × 16 matrices described in O *et al.* 2019.

## 4. Results   

**Figure 6:**
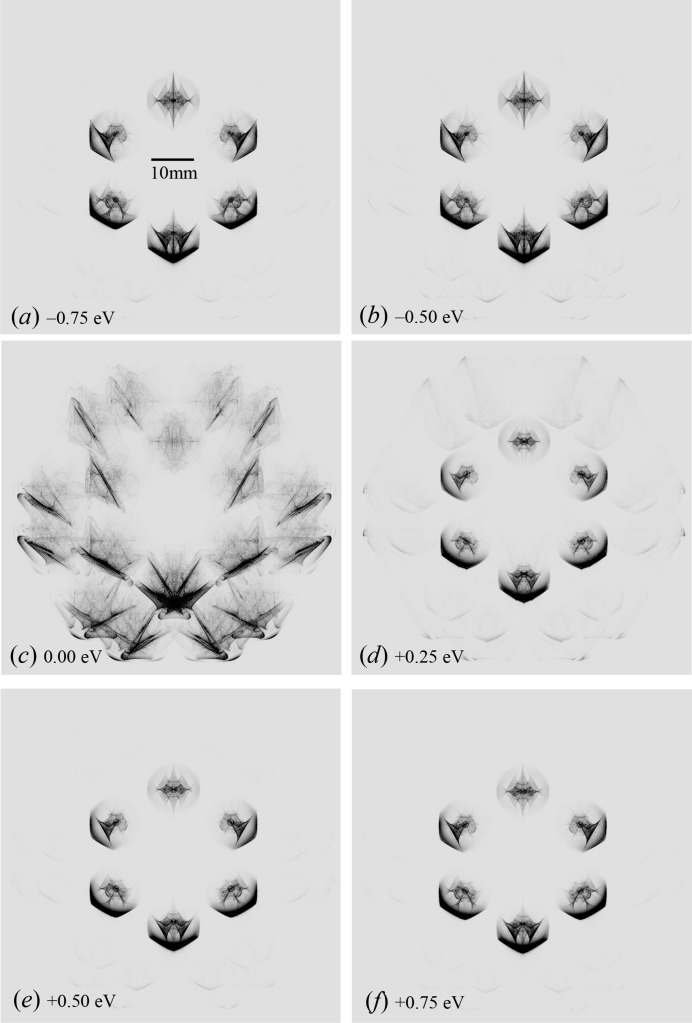
When the photon energy is 21.9843937 keV, the inner six and outer 12 reciprocal-lattice nodes (see Fig. 4 ▸) can be present simultaneously on an identical surface of the Ewald sphere. The deviations of photon energies from *E*_0_ (= 21.98440 keV) were assumed to be −0.75, −0.50, 0.00, +0.25, +0.50 and +0.75 eV for (*a*), (*b*), (*c*), (*d*), (*e*) and (*f*), respectively.

Fig. 6 ▸(*c*) shows the E-L&FFT simulated result with a photon energy of 21.98440 keV. In this figure, X-ray diffraction intensities due to the outer 12 reciprocal-lattice nodes on the blue circle in Fig. 4 ▸ are as strong as the inner six diffraction patterns that are substantially different from the experimentally obtained topograph in Fig. 3 ▸(*a*). However, the outer 12 topograph patterns are almost unobservable when the energy deviation from 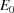 (= 21.98440 keV) is over 0.50 eV. Thus the present authors conclude that the photon energy of the synchrotron X-rays used in the present experiment was ∼21.98415 keV with which Fig. 3 ▸(*a*) was obtained.

**Figure 7:**
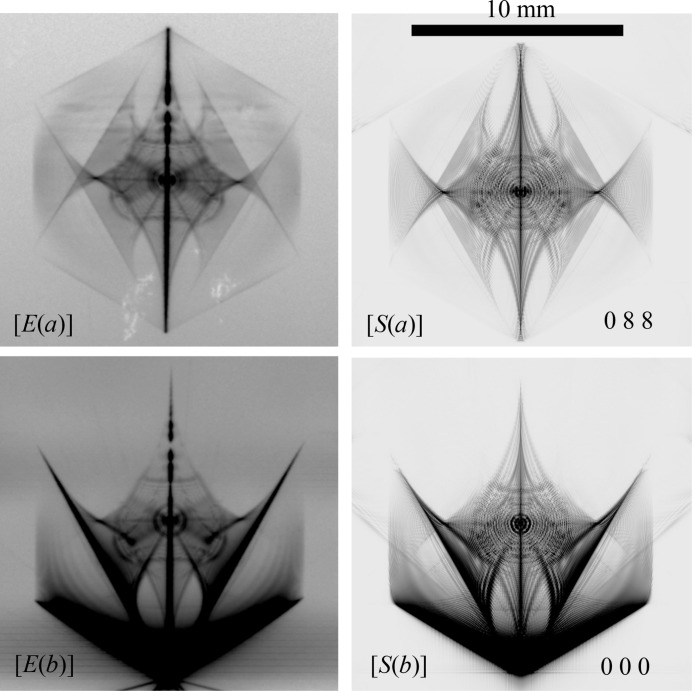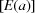 and 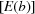 are enlargements of 088 TR and 000 FD X-ray patterns of Fig. 3 ▸(*a*) obtained experimentally. 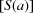 and 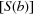 are enlargements of 088 TR and 000 FD X-ray patterns of Fig. 3 ▸(*b*) obtained by the E-L&FFT simulation.

Fig. 7 ▸ shows enlargements of 088 TR and 000 FD images from Figs. 3 ▸(*a*) and 3 ▸(*b*). There is remarkable consistency between the experimentally obtained and the E-L&FFT simulated images.

## 5. Discussion   

**Figure 8:**
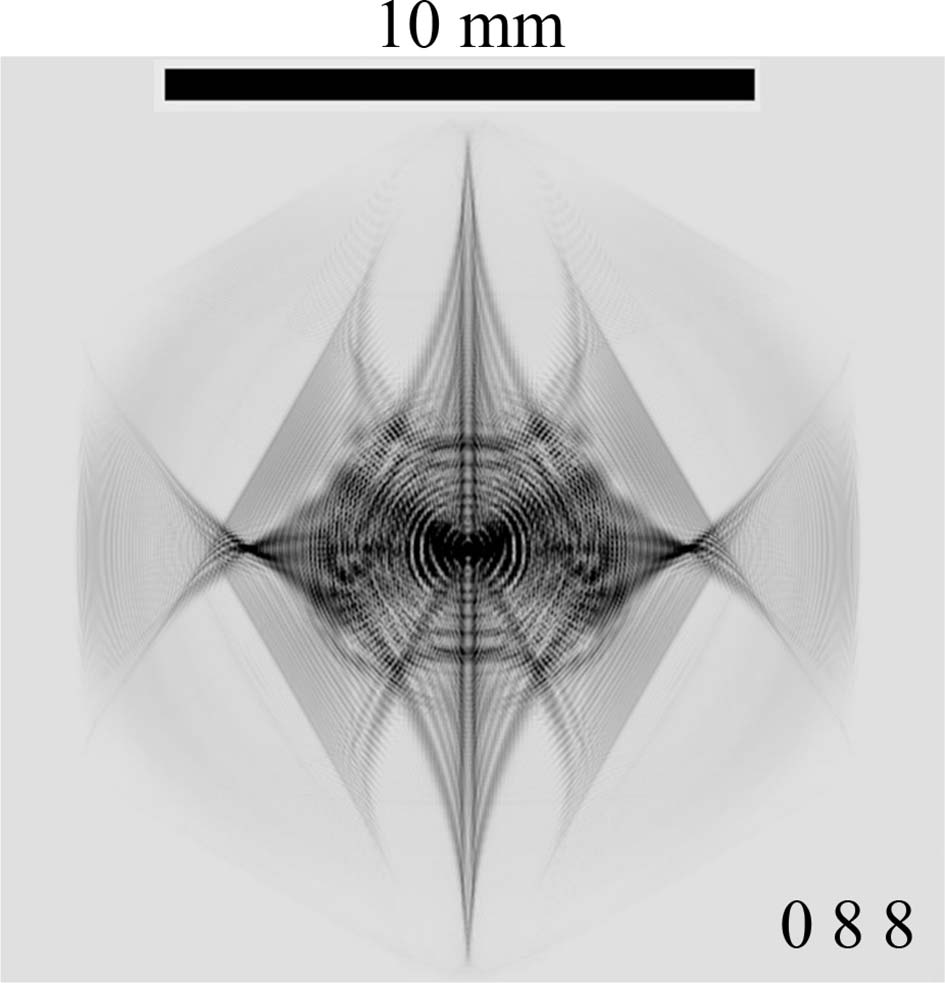
E-L&FFT simulated 088 TR topograph images with a photon energy of 21.98415 keV under an assumption of the six-beam case; here, the 000 FD, 440, 484, 088, 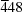 and 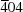 TR X-rays are strong by neglecting the outer 12 beams. An evident discrepancy is observed between this figure and Fig. 7 ▸ 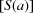.

Fig. 8 ▸ shows an image of 088 TR X-rays obtained by the E-L&FFT simulation omitting the presence of the outer 12 reciprocal-lattice nodes. The assumed photon energy was identical to that in Fig. 7 ▸ [*S*(*a*)] (21.984150 keV). The vertical centre line in Fig. 8 ▸ was divided into two lines, whereas only one vertical line was observed in Fig. 7 ▸ [*S*(*a*)]. Further, an evident difference in the central part was observed between Fig. 7 ▸ [*S*(*a*)] and Fig. 8 ▸. It has been clarified that the presence of the outer 12 reciprocal-lattice nodes affected the features of the inner six diffraction patterns.

Incidentally, referring to Fig. 5 ▸, let another Laue point 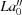 be defined at a position on 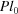 such that it is not far from *La* and 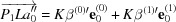. Further, let 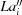 be defined such that 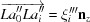 on 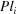 whose distance from 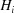 is *K* (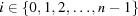). By replacing 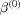, 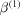 and 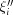 in equations (9) and (10) with 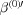, 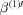 and 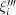, respectively, the following equation is obtained: 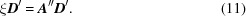
Here, 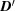 is a 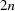-order column vector and 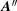 is a 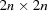 matrix whose element in the *p*th row 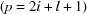 and *q*th column 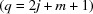 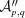 is given by 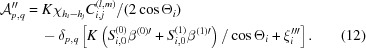
This way of defining 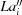, 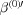, 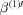, 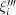 and 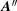 and equations (11) and (12) are more general than equations (9) and (10). Even when *La* cannot be defined as shown in Fig. 5 ▸, the eigenvalue problem represented by (11) can be solved. Then, the intensity distribution of reflected X-rays can be calculated with the E-L&FFT method when a pinhole X-ray beam is incident on an arbitrary position of the surface of the crystal. This is also the case for a crystal as shown in Fig. 9 of O *et al.* 2019 owing to the description given therein. The total intensities of X-rays reflected from the crystal completely bathed in the incident X-rays can be calculated by incoherently superposing the pinhole topograph intensities with the incident position two-dimensionally scanned over the incident side of the crystal.

## 6. Summary   

In the present non-coplanar 18-beam case, the 18 reciprocal-lattice nodes are on two circles, drawn in red and blue in Fig. 4 ▸. The most important aspect of the present work is that a non-coplanar *n*-beam case for 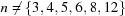 was computer simulated using the E-L&FFT method and was reasonably consistent with the experimentally obtained result. The constraint that 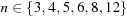 has been originally placed such that *n* reciprocal-lattice nodes are on a circle in the reciprocal space. In the case of protein crystals as shown in Fig. 1 ▸(*b*), the situation where a large number of reciprocal-lattice nodes are simultaneously present in the vicinity of the surface of the Ewald sphere cannot be circumvented.

However, the constraint on *n* has been removed completely from the *n*-beam E-L&FFT method to calculate the X-ray diffraction intensities. *N* is the number of reciprocal-lattice nodes present in the vicinity of the surface of the Ewald sphere whose presence should be considered. Another difficulty caused by the complex shape of the crystal has also been overcome with the description in O *et al.* 2019. Thus, the present authors could calculate the intensities of X-ray diffraction spots as shown in Fig. 1 ▸(*b*) under the assumption that the crystal is perfect.

## Figures and Tables

**Table 1:** The position of the point *La* whose distance from the *i*th-numbered reciprocal-lattice nodes 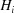 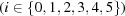 is an identical length *K*, calculated for a photon energy of 22.0 keV The Miller indices were 000, 440, 484, 088, 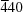 and 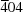. 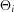 (°) 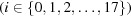 is the angle spanned by the directions of 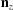 and 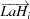. 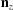 is a unit vector in the direction of 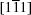 (downward surface normal). When 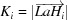, 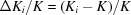. 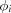 is the inclination angle of 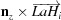 from 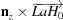. 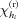 and 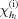 are, respectively, the real and imaginary parts of the 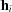
th-order Fourier coefficient of the electric susceptibility as calculated using *XOP 2.3* (Sanchez del Rio & Dejus, 1998 ▸) for a photon energy of 22.0 keV. Identical values of 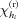 and 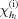 were used for all the simulations shown in Figs. 3 ▸, 6 ▸ and 7 ▸ because the energy differences from 22.0 keV are negligible.

Ordinal number *i*	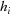	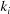	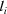	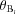 (°)	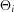 (°)	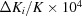	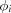 (°)	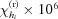	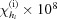
0	0	0	0	0.0000	35.9750	0.0000	0.0000	−2.004400	−0.625153
1	4	4	0	17.0806	35.9750	0.0000	60.0000	−0.773093	−0.550274
2	4	8	4	30.5793	35.9750	0.0000	120.0000	−0.296936	−1.136870
3	0	8	8	35.9750	35.9750	0.0000	180.0000	−0.214413	−0.375281
4	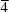	4	8	30.5793	35.9750	0.0000	240.0000	−0.296936	−0.426348
5	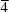	0	4	17.0806	35.9750	0.0000	300.0000	−0.773093	−0.550274
6	1	1	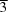	9.9161	50.9503	−1.5751	19.1066	−0.784785	−0.423082
7	3	3	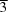	15.6521	50.9503	−1.5751	40.8934	+0.586813	+0.396937
8	5	7	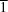	26.7218	50.9503	−1.5751	79.1066	+0.285189	+0.327801
9	5	9	1	32.4855	50.9503	−1.5751	100.8933	−0.183684	−0.288538
10	3	11	5	40.2726	50.9503	−1.5751	139.1066	+0.128538	+0.238282
11	1	11	7	42.7632	50.9503	−1.5751	160.8934	+0.116815	+0.223557
12	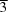	9	9	42.7632	50.9503	−1.5751	199.1066	−0.116815	−0.223557
13	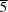	7	9	40.2726	50.9503	−1.5751	220.8934	+0.128538	+0.238282
14	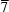	3	7	32.4855	50.9503	−1.5751	259.1066	+0.183684	+0.288538
15	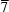	1	5	26.7218	50.9503	−1.5751	280.8934	−0.285189	−0.327801
16	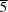	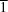	1	15.6521	50.9503	−1.5751	319.1066	+0.586813	+0.396937
17	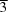	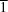	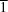	9.9161	50.9503	−1.5751	340.8934	−0.784785	−0.423082

**Table 2:** Values of 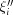 for 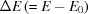 are −0.75, −0.50, −0.25, 0.00, +0.25, +0.50 and +0.75 eV, where 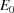 = 21.98440 keV

Fig. No. of the simulation	Photon energy *E* (keV)	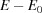 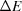 (eV)	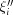 (m 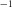 )
Fig. 6 ▸(*a*)	21.98365	−0.75	+2.11782 × 10^5^
Fig. 6 ▸(*b*)	21.98390	−0.50	+1.40582 × 10^5^
Fig. 3 ▸(*b*)	21.98415	−0.25	+0.69386 × 10^5^
Fig. 6 ▸(*c*)	21.98440	0.00	0.01805 × 10^5^
Fig. 6 ▸(*d*)	21.98465	+0.25	−0.72993 × 10^5^
Fig. 6 ▸(*e*)	21.98490	+0.50	−1.44176 × 10^5^
Fig. 6 ▸(*e*)	21.98515	+0.75	−2.15356 × 10^5^
